# Expression of pathogenesis-related proteins in transplastomic tobacco plants confers resistance to filamentous pathogens under field trials

**DOI:** 10.1038/s41598-019-39568-6

**Published:** 2019-02-26

**Authors:** Noelia Ayelen Boccardo, María Eugenia Segretin, Ingrid Hernandez, Federico Gabriel Mirkin, Osmani Chacón, Yunior Lopez, Orlando Borrás-Hidalgo, Fernando Félix Bravo-Almonacid

**Affiliations:** 1 Laboratorio de Biotecnología Vegetal, Instituto de Investigaciones en Ingeniería Genética y Biología Molecular “Dr. Héctor N. Torres” (INGEBI-CONICET), (C1428ADN) Ciudad Autónoma de Buenos Aires, Argentina; 2 Departamento de Fisiología, Biología Molecular y Celular, Facultad de Ciencias Exactas y Naturales, Universidad de Buenos Aires, (C1428EGA) Ciudad Autónoma de Buenos Aires, Argentina; 3 Centro de Ingeniería Genética y Biotecnología (CIGB), (10600) La Habana, Cuba; 4 Shandong Provincial Key Laboratory of Microbial Engineering, School of Biotechnology, Qi Lu University of Technology, Jinan, (250353) P.R. China; 5 Departamento de Ciencia y Tecnología, Universidad Nacional de Quilmes, Bernal, Buenos Aires, (B1876BXD) Argentina

**Keywords:** Molecular engineering in plants, Molecular engineering in plants

## Abstract

Plants are continuously challenged by pathogens, affecting most staple crops compromising food security. They have evolved different mechanisms to counterattack pathogen infection, including the accumulation of pathogenesis-related (PR) proteins. These proteins have been implicated in active defense, and their overexpression has led to enhanced resistance in nuclear transgenic plants, although in many cases constitutive expression resulted in lesion-mimic phenotypes. We decided to evaluate plastid transformation as an alternative to overcome limitations observed for nuclear transgenic technologies. The advantages include the possibilities to express polycistronic RNAs, to obtain higher protein expression levels, and the impeded gene flow due to the maternal inheritance of the plastome. We transformed *Nicotiana tabacum* plastids to co-express the tobacco PR proteins AP24 and β-1,3-glucanase. Transplastomic tobacco lines were characterized and subsequently challenged with *Rhizoctonia solani*, *Peronospora hyoscyami* f.sp. *tabacina* and *Phytophthora nicotianae*. Results showed that transplastomic plants expressing AP24 and β-1,3-glucanase are resistant to *R*. *solani* in greenhouse conditions and, furthermore, they are protected against *P*.*hyoscyami* f.sp. *tabacina* and *P*. *nicotianae* in field conditions under high inoculum pressure. Our results suggest that plastid co- expression of PR proteins AP24 and β-1,3-glucanase resulted in enhanced resistance against filamentous pathogens.

## Introduction

Plant diseases affect most staple crops decreasing productivity worldwide and severely compromising food security^1–3^. Management of diseases caused by fungal and oomycete pathogens is mainly achieved by applications of chemicals that cost hundreds of millions of dollars annually, some of which are likely to be banned in the near future. Moreover, due to rapid evolution of the pathogen population, resistant strains could be selected^4,5^. The situation is expected to get worse, taking into account the current rate of human population growth, the effect of climate change, the movement of contaminated material, the prevalence of monocultures and the rise in overall cultivated area^6^.

Plants have evolved different mechanisms to counterattack pathogen infection, including several layers of constitutive and inducible defences^7,8^. For induction to occur, plants have evolved immune receptors that activate effective defense responses upon detection of pathogens molecules. Plant immunity is triggered by the detection of microbe-, pathogen- or damage-associated molecular patterns (MAMPs, PAMPs or DAMPs respectively) that lead to pattern-triggered immunity (PTI), and by the detection of effectors that activate effector-triggered immunity (ETI)^7,9^. PAMP and effector recognition trigger local signalling events including ion fluxes, production of reactive oxygen species (ROS) and induction of protein kinases, leading to the production of phytohormones, phytoalexins, phenolic compounds and pathogenesis-related (PR) proteins^10^, ultimately leading to a hypersensitive response (HR), a type of programmed cell death that takes place at the site where pathogen attempts invasion^11^). Salicylic acid (SA) and jasmonate (JA) are recognized as the most important hormones for plant immune responses, with SA and JA biosynthesis and signalling being historically related to defense against biotrophic or necrotrophic pathogens, respectively^12,13^. SA accumulation and the coordinated activation of PR genes are necessary for the establishment of Systemic Acquired Resistance (SAR) in tissues distant from the primary infection site^14,15^.

PR proteins comprise a group of inducible and functionally diverse proteins that accumulate in response to pathogen attack. These proteins have been implicated in active defense, potentially restricting pathogen development and spread^10,16,17^. Transcripts corresponding to PR proteins accumulate within minutes to hours of PTI and ETI induction, the expression of most of them being regulated by SA. To date, seventeen families of PR proteins (PR-1 to PR-17) have been described in most plant species^10^. Regarding their role in defense, PR proteins can directly affect pathogen integrity, and/or generate signal molecules through their enzymatic activity that act as elicitors to induce other plant defense related pathways^10,16,17^. Most PR protein families include members whose activities are coherent with a role in plant defense against fungal and/or oomycete pathogens: β-1,3-endoglucanases (PR-2), endochitinases (PR-3, 4, 8 and 11), thaumatin-like proteins (PR-5), defensins (PR-12), thionins (PR-13) and lipid transfer proteins (PR-14)^10^.

A sustainable method for disease management is growing disease resistant varieties that provide durable resistance^18^. For several decades now, breeding strategies transferring mainly monogenic resistance from wild relatives to crops has proved to be a useful strategy to deploy resistant varieties in the field, although in most cases resistance was not long-lasting due to pathogen evolution^19,20^. Stacking of major resistance (*R*) genes can help to overcome this limitation, but even when accelerated by marker-assisted selection, this strategy comes with certain difficulties, particularly for highly polyploid species and/or species who reproduce mainly by clonal propagation, together with the limitation for discovering new *R* genes in sexually compatible species^20^. Genetic engineering has provided a powerful tool to overcome many limitations of conventional breeding strategies, prompted by a more thorough understanding of the molecular basis of disease-resistance in plants^21–23^. As a general rule, the deployment of genetic engineering approaches that involve the expression of two or more antimicrobial gene products, including PR proteins, in a specific crop should provide more effective and broad-spectrum disease control than the single-gene strategy^24–27^. The efficiency of PR genes in transgenic approaches to obtain pathogen resistance is well documented (for a review see^17,28,29^). Numerous transgenic plant developments have been reported in which varying degrees of protection against specific fungal and/or oomycete pathogens have been achieved. However, in many cases the levels of associated resistance were insufficient for breeding purposes^25,30^. Moreover, constitutive expression of PR proteins can result in a lesion mimic phenotype (i.e., the spontaneous formation of lesions resembling HR lesions in the absence of a pathogen) that can arise as an undesirable consequence^31–33^. In order to be adopted by farmers, strategies to deploy disease resistance, should be able to control specific diseases without affecting crops yield or quality^34^.

Plastid genome (plastome) transformation could overcome limitations mentioned above. This alternative approach enables the accumulation of higher levels of heterologous proteins enclosed in the organelle, with reported values of up to 51% and even 70% of total soluble protein (TSP) in *Nicotiana tabacum* plants^35,36^. Other advantages over nuclear genome transformation include the absence of positional effects and silencing, as well as the reduced risk of transgene transfer to compatible wild relatives as a consequence of the maternal inheritance of plastids in many crops^37,38^. Transplastomic plants have been developed for multiple purposes, including but not limited to, molecular farming, metabolic engineering, and phytoremediation^39^. Regarding biotic stress resistance, transplastomic plants accumulating long double-stranded RNA in their plastids have successfully controlled insects^40^. Promising results regarding resistance to fungal pathogens published to date include the transformation of the chloroplast genome to accumulate MSI-99 (a synthetic antimicrobial peptide)^41,42^, and the expresion of chloroperoxidase from *Pseudomonas pyrrocinia*^43^, both cases resulting in strong inhibition of spore germination and/or growth in *Aspergillus flavus*, *Fusarium moniliforme*, and *Verticillium dahliae* (*in vitro* assays), and limitation of lesion size after inoculation of transplastomic plants with *Alternaria alternata*^43^ or *Colletotrichum destructivum*^41^. To date, there are no reports demonstrating whether plastid transformation is a suitable strategy to control oomycete pathogens. This particular phylogenetic group includes *Phytophthora sp*., one of the most destructive genera, whose member species are responsible for severe economic losses on crops worldwide, as well as environmental damage in natural ecosystems^44,45^.

In this work we have tested whether transformation of the plastid genome to express PR proteins in the chloroplast stroma protects tobacco against three dangerous filamentous pathogens with different lifestyles: a necrotrophic fungal pathogen (*Rhizoctonia solani*), a hemibiotrophic oomycete (*Phytophthora nicotianae*) and an obligate biotrophic oomycete (*Peronospora hyoscyami* f.sp. *tabacina*). We chose to co-express a *N*. *tabacum* basic β-1,3-glucanase (an antifungal hydrolase associated with SAR response in plants belonging to the PR-2 family), and the *N*. *tabacum* osmotin AP24 (PR-5 family), both proven to effectively control fungal and oomycete pathogen infection in different transgenic crops^17,29,46^. Protection against *R*. *solani* was assessed in greenhouse conditions while field trials were carried out for *P*. *hyoscyami* f. sp. *tabacina* and *P*. *nicotianae*. Transplastomic plants expressing both AP24 and β-1,3-glucanase are highly resistant to *R*. *solani* and moreover, are protected against *P*. *hyoscyami* f. sp. *tabacina* and *P*. *nicotianae* in field conditions under high inoculum pressure. Our results indicate that co-expression of AP24 and β-1,3-glucanase in the chloroplast enhanced resistance against filamentous pathogens.

## Results

### Production of transplastomic *N*. *tabacum* expressing AP24 and β-1,3-glucanase

**Figure 1:**
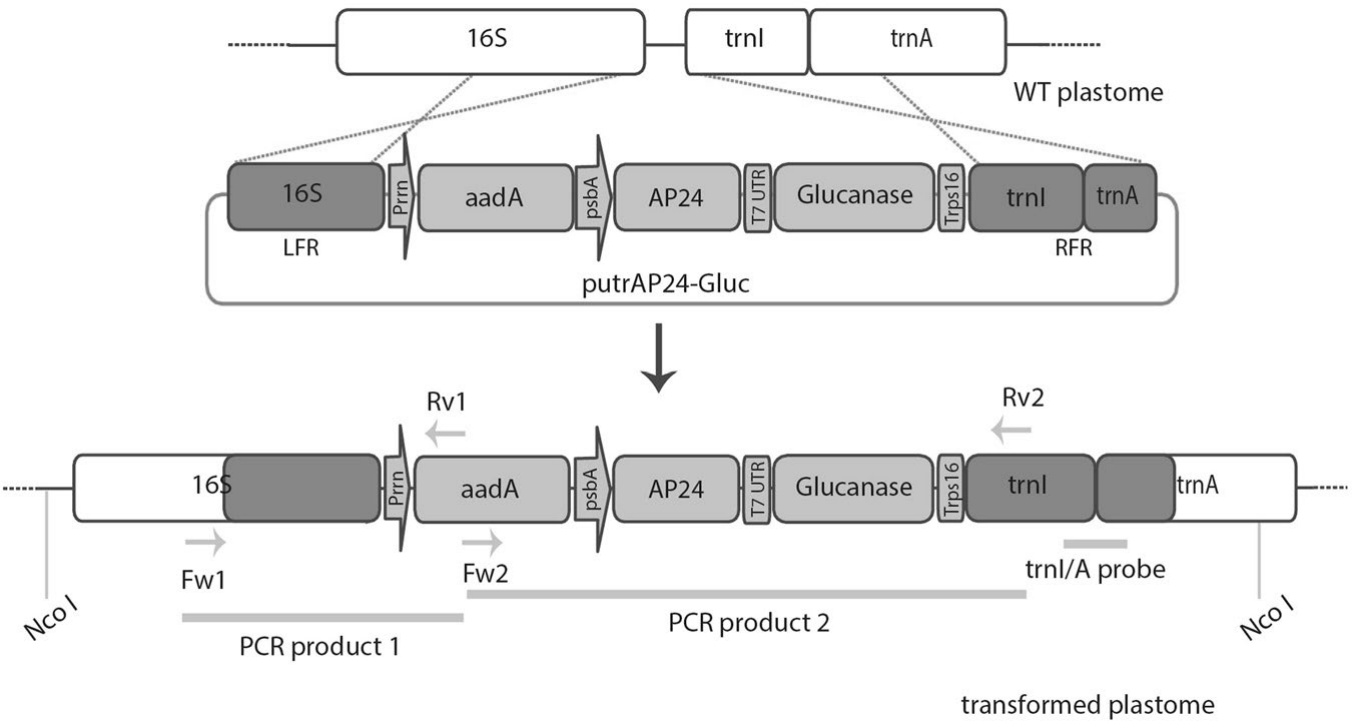
Design of the plastid transformation vector putrAP24-Gluc. Vector putrAP24-Gluc includes AP24 and β-1,3-glucanase coding sequences downstream promoter and 5′ untranslated region of *psbA* (psbA). The 5′ untranslated region from T7 phage gene 10 (T7 UTR) provides a functional ribosome binding site (RBS) for β-1,3-glucanase. The *aadA* sequence is under the control of the *rrn* operon promoter (Prrn). We included left flanking region (LFR) and right flanking region (RFR) as recombinogenic sequences. trnI/A: probe used in Southern blot assays. Primers used in PCR are indicated by arrows.

To obtain transplastomic tobacco plants (*N*. *tabacum* L. cv. Petit Havana, WT) expressing AP24 and β-1,3-glucanase, we designed a vector designated putrAP24-Gluc (Fig. 1). This vector is derived from the pBSW-utr vector and contains *N*. *tabacum* sequences to target the integration of transgenes into the intergenic region between the *16S* and *trnI* genes by homologous recombination^47^. AP24 and β-1,3-glucanase coding sequences were assembled to generate a dicistronic RNA, where the second cistron is preceded by the 5′-untranslated region (5′-UTR) from gene 10 of the T7 phage (T7g10)^48^ (Fig. 1). putrAP24-Gluc was used to transformed *N*. *tabacum* plastid genome by particle bombardment of tobacco leaves.

**Figure 2:**
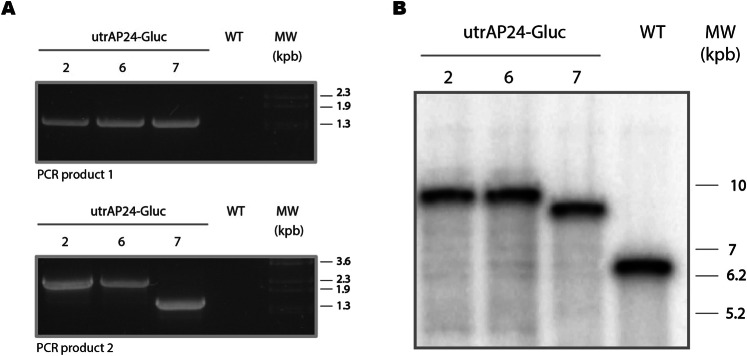
Analysis of transgene integration and homoplasmic state. (**A**) Transplastomic nature of the plants was confirmed by PCR analysis, using Fw1 and Rv1 (above) and Fw2 and Rv2 primers (below). WT: wild-type tobacco plant. MW: Lambda BstEII marker. (**B**) Homoplasmy and stable integration of transgenes was evaluated by Southern Blot with a trnI/A probe (wild-type plastome: 6, 4 kbp, transformed plastome: 9, 6 kbp). Bands corresponding to a 1-kb DNA marker are indicated on the right.

We confirmed the transplastomic nature of the regenerated shoots by PCR, using the Fw1 and Rv1 primers (Fig. 1). Three independent lines (utrAP24-Gluc 2, 6 and 7) showing integration of the construct at the desired location in the plastid genome were obtained (Fig. 2A). Another PCR assay was performed using the Fw2 and Rv2 primers to confirm the integrity of the inserted transgenes. This assay showed a fragment of the expected size in utrAP24-Gluc 2 and 6 lines, but not in utrAP24-Gluc 7 (Fig. 2A). These lines were subjected to additional rounds of regeneration under selective pressure to achieve homoplasmy. Mature plants were transferred to soil for further characterization.

### Molecular and phenotypic characterization of transplastomic plants

To evaluate the stable integration of the transgenes in the plastid genome, and the homoplasmy of transplastomic lines, we analyzed by Southern blot the total leaf DNA digested with NcoI. We used a trnI/A probe that allows distinguishing the presence of wild-type plastome copies (6.4 Kbp fragment) from transformed plastome copies (9.6 Kbp fragment) (Fig. 1). Only the 9,6 Kbp transgenic DNA fragment was detected in utrAP24-Gluc lines 2 and 6, with no visible band corresponding to wild-type plastome (Fig. 2B). In the case of utrAP24-Gluc line 7, a band of smaller size was observed (Fig. 2B), in agreement with the results observed by PCR (Fig. 2A). The results confirmed the transplastomic nature of all the utrAP24-Gluc lines, as well as their homoplasmic state.

**Figure 3:**
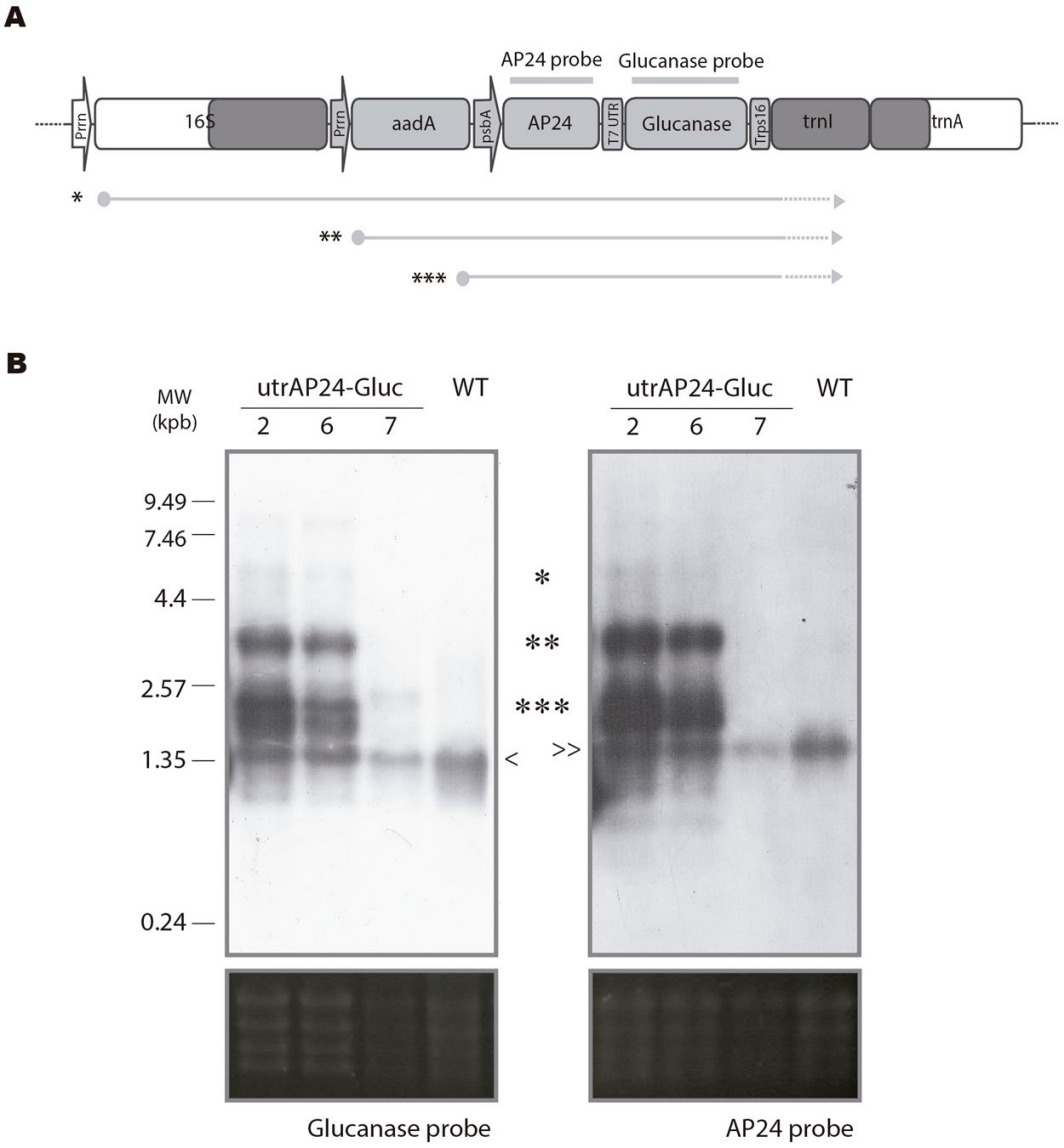
Characterization of transcripts containing the glucanase and AP24 sequence of transplastomic plants. (**A**) Physical map showing expected RNA transcripts from each promoter in transplastomic plants. (*) polycistronic read-through transcript (4.5 kb), (**) tricistronic transcript (2.9 kb). (***), dicistronic transcript (1.8 kb). AP24 probe and Glucanase probe are indicated. (**B**) Northern blot using glucanase (left panel) and AP24 (right panel) probes showing transcript generation. *Policistronic, **tricistronic, ***dicistronic, and endogenous glucanase and AP24 mRNAs (<and ≫, respectively). Total rRNA visualized under UV light was included as loading control.

To evaluate transcript profiles in the transplastomic plants, we performed a northern blot assay probed with sequences homologous to AP24 and glucanase. As observed in Fig. 3B, a band corresponding to the endogenous glucanase and AP24 mRNAs could be observed in all analysed samples (< and ≫, respectively). Also, three major transcripts from transplastomic origin could be detected in utrAP24-Gluc 2 and 6 transplastomic lines. The dicistronic transcript (***) transcribed from the *psbA* promoter was the most abundant, in accordance with previous reports^36,47^. The *rrn* promoter included to control *aadA* and AP24/Glucanase expression was responsible for a tricistronic transcript (**). We could also detect a larger transcript originated as a consequence of read-through transcription from an endogenous *rrn* promoter (*, Fig. 3A). Expected transcript sizes were approximately 1.8 kb for the dicistronic, 2.9 kb for the tricistronic and 4.5 kbp for the largest transcript, in agreement with their electrophoretic mobility relative to the mobility of rRNA 25S and 16S transcripts (3.7 and 1.5 kb, respectively)

We decided not to include the utrAP24-Gluc 7 line in the following experiments, since it presents unexpected band profiles in PCR, Southern blot (Fig. 2), and northern blot (Fig. 3) assays.

**Figure 4:**
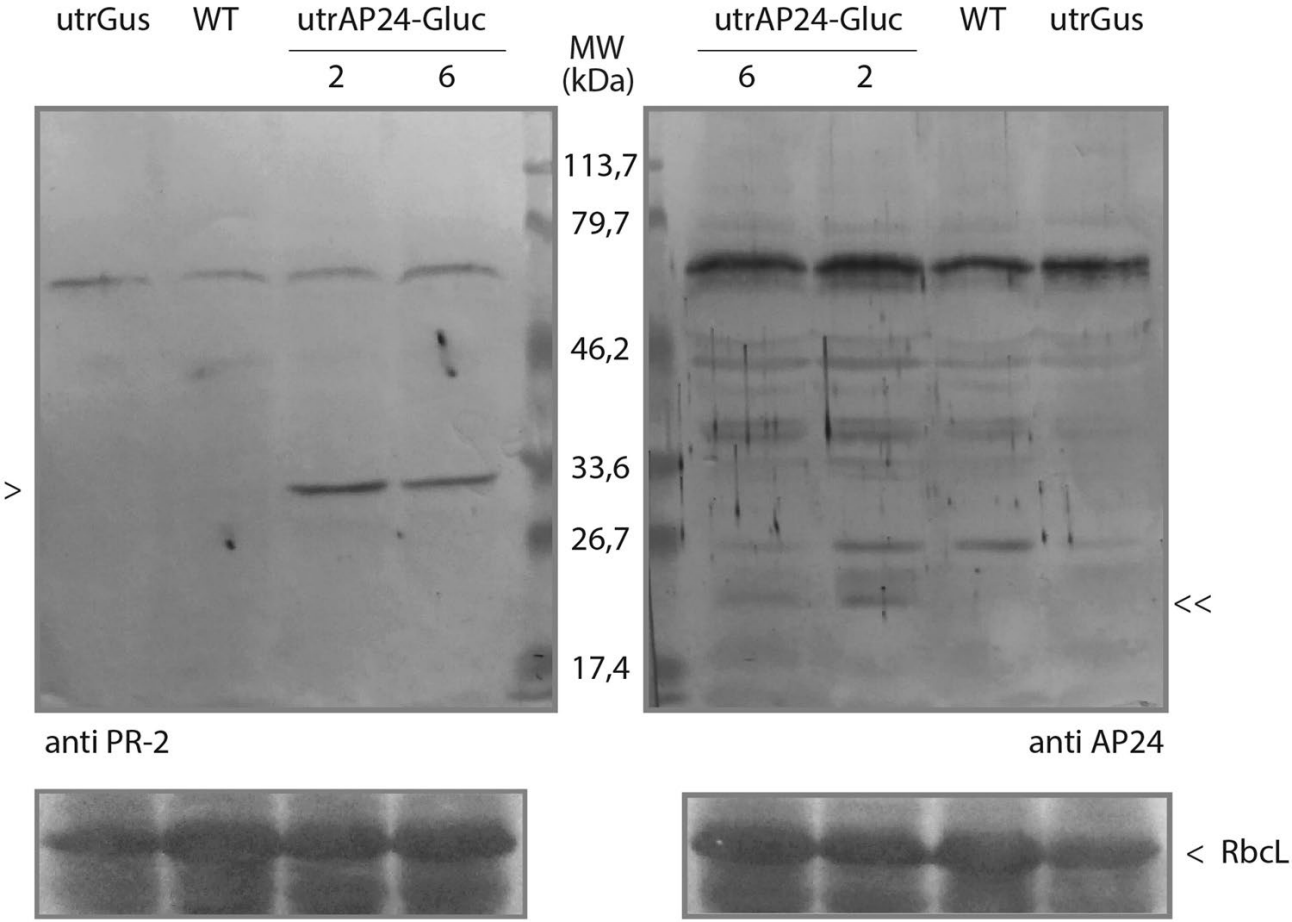
Expression of AP24 and β-1,3-glucanase in transplastomic plants. For western blot assay, lanes were loaded with total protein extracts obtained from 4 mg of fresh leaf tissue from wild-type (WT) or transplastomic plants (utrGus, utrAP24-Gluc 2 and 6). The position of molecular weight marker (Bio-Rad Prestained SDS-PAGE Standards, broad range) is indicated on the middle. β-1,3-glucanase and AP24 positions are indicated with < and ≫, respectively. The band corresponding to the large subunit of RuBisCO (RbcL) after Ponceau Red staining was included as the loading control.

To determine β-1,3-glucanase and AP24 protein accumulation, we analysed utrAP24-Gluc 2 and 6 transplastomic lines and control plants protein extracts by western blot (Fig. 4). As control plants we included the transplastomic line utrGus^47^ and wild-type tobacco plants. Both proteins could be detected in utrAP24-Gluc 2 and 6 plants, indicating that overexpression of these PR proteins can be achieved by plastid genome transformation.

**Figure 5:**
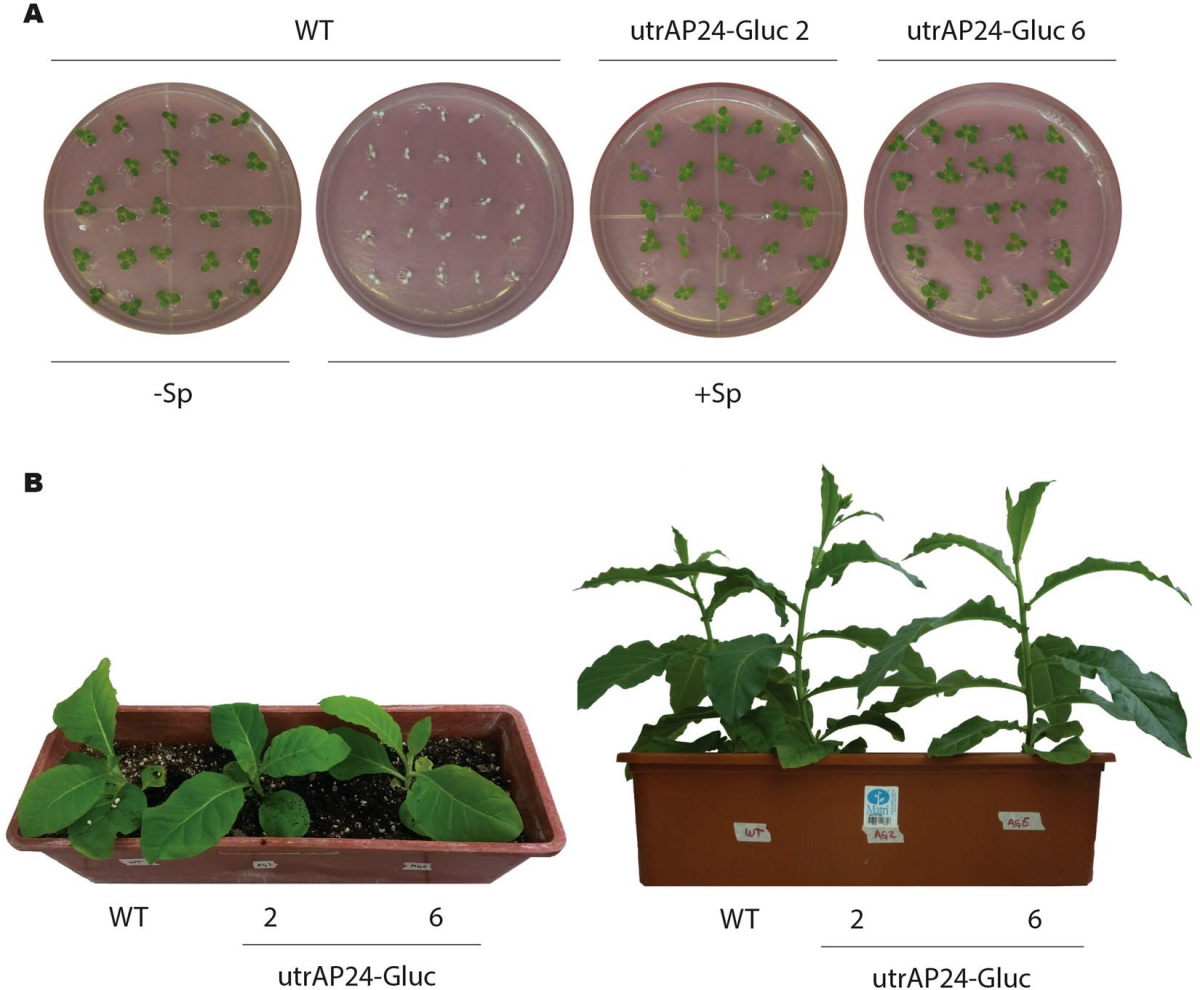
Phenotypes of transplastomic tobacco plants. (**A**) Seeds from wild-type (WT) or transplastomic plants (utrAP24-Gluc 2 and 6) were germinated on synthetic medium with (+Sp) or without (−Sp) spectinomycin (500 mg/L). Phenotypes were recorded 15 days after sowing the seeds. (**B**) Phenotypes of wild-type (left) and transplastomic plants utrAP24-Gluc 2 (center) and utrAP24-Gluc 6 (right) recorded 8 and 12 weeks after sowing the seeds.

To further confirm homoplasmy, and in order to evaluate the phenotype of utrAP24-Gluc transplastomic plants, we germinated seeds in spectinomycin containing media. As observed in Fig. 5A, all the seeds from lines utrAP24-Gluc 2 and 6 germinated in the presence of spectinomycin and presented a normal phenotype that was maintained throughout plant development. As observed in Fig. 5B, mature transplastomic plants were indistinguishable from their non-transformed counterparts.

### Enhanced disease resistance of transplastomic tobacco plants to *R*. *solani* under greenhouse conditions

**Figure 6:**
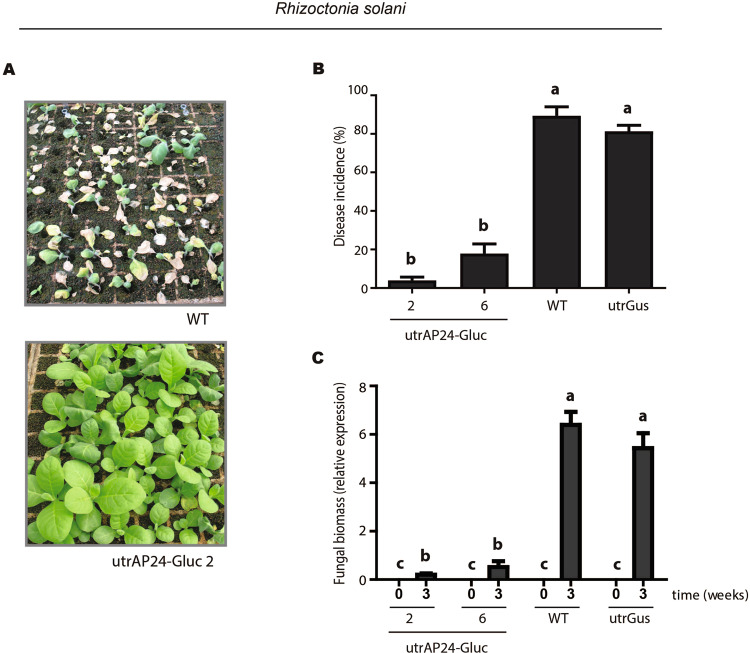
Greenhouse evaluation of transgenic tobacco plants for resistance against *Rhizoctonia solani*. (**A**) Phenotype of wild-type (above) and utrAP24-Gluc 2 (below) tobacco plants inoculated with *R*. *solani* at 3 weeks post-inoculation (wpi). (**B**) Evaluation of disease incidence of *R*. *solani* in transplastomic tobacco plants. (**C**) Quantitative real time PCR to measure *R*. *solani* (fungal biomass) growth in transgenic plants at 3 wpi. Bars represent mean values (N = 90; ±SD). Different letters within the columns indicate significant differences (*p* < 0.0001).

To evaluate if expression of AP24 and β-1,3-glucanase coding sequences from the chloroplast genome can protect tobacco plants against filamentous pathogens, we challenged transplastomic tobacco lines utrAP24-Gluc 2 and 6 with the necrotrophic soil-borne fungal pathogen *R*. *solani*, the causal agent of tobacco leaf spot and root rot, under greenhouse conditions^49^. We could observed for utrGus and wild-type tobacco plants the characteristic symptoms caused by *R*. *solani* (as previously described^49^), consisting on small stem water-soaked lesions that rapidly became brown and sunken, predominantly at the lower part of the stem, near the soil (Fig. 6A). As disease progressed, these lesions expanded throughout the stems and the tissue turned brown and finally died. Disease incidence was high on utrGus and wild-type plants but was significantly lower in utrAP24-Gluc 2 and 6 transplastomic lines (Fig. 6B). Symptom development was in accordance with fungal biomass quantification in the colonized roots of control and utrAP24-Gluc 2 and 6 transplastomic plants (Fig. 6C). These results indicate that chloroplast genome transformation with AP24 and β-1,3-glucanase coding sequences protects tobacco plants from a necrotrophic fungal pathogen infection.

### Enhanced disease resistance of transplastomic tobacco plants to *P*. *nicotianae* and *P*. *hyoscyami* f. sp. *tabacina* infection under field conditions

**Figure 7:**
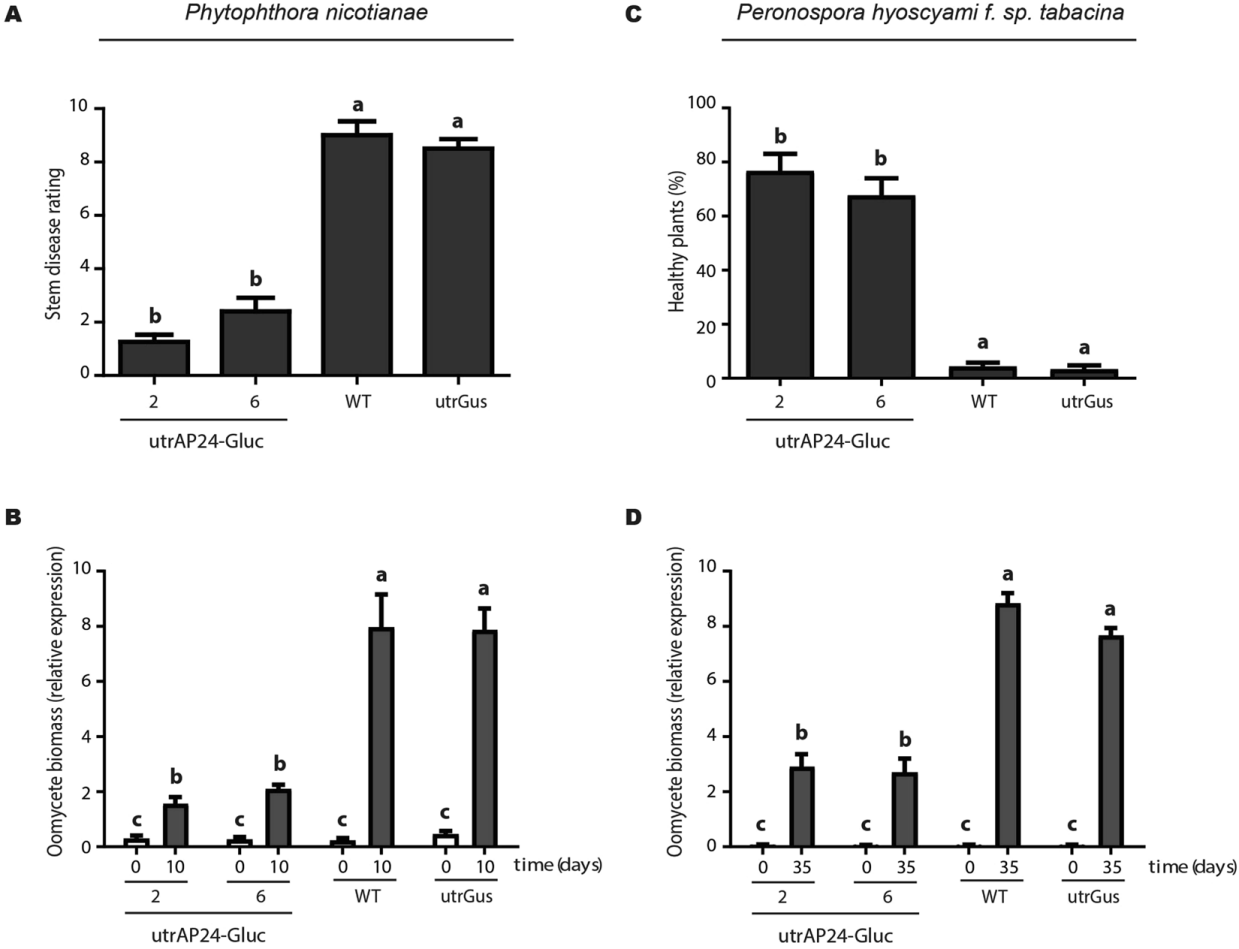
Field evaluation of transgenic tobacco plants for resistance against *Phytophthora nicotianae* and *Peronospora hyoscyami* f. sp. *tabacina*. Quantification of stem disease rating (**A**) and *P*. *nicotianae* biomass (**B**) in transgenic tobacco plants planted in a soil with a high inoculum of *P*. *nicotianae* at 10 days after planting. Quantification of healthy plants (**C**) and *P*. *hyoscyami* f. sp. *tabacina* biomass (**D**) in transgenic tobacco plants planted in an area with high incidence of *P*. *hyoscyami* f. sp. *tabacina* at 35 days after planting. Pathogen biomass measured by quantitative real time PCR is shown as relative expression. Bars represent mean values (N = 90; ± SD). Different letters within the columns indicate significant differences (*p* < 0.0001).

To evaluate the performance of utrAP24-Gluc transplastomic plants in natural infections, we designed field trials that were carried out in experimental fields located at the Center of Biotechnology and Genetic Engineering, Havana, Cuba, as previously described^49^. Under field conditions the transplastomic tobacco lines utrAP24-Gluc 2 and 6 also showed enhanced resistance to the hemibiotrophic oomycete *P*. *nicotianae*, responsible for the black shank disease. At five days post-planting utrGus and wild-type plants presented slight disease symptoms whereas no symptoms were observed on the utrAP24-Gluc 2 and 6 transplastomic plants. Five days later, we observed leaf wilting and stem rot (considered to be severe disease symptoms^49^) in the utrGus and wild-type plants. On the other hand, both transplastomic lines expressing AP24 and β-1,3-glucanase combination remained healthy, indicating a high level of resistance to *P*. *nicotianae* (Fig. 7A). Furthermore, as quantification of oomycete biomass by real-time PCR, confirmed that *P*. *nicotianae* gradually increased in utrGUS and wild-type plants while only a minor increase in pathogen biomass was observed in utrAP24-Gluc 2 and 6 transplastomic plants (Fig. 7B).

To analyze the performance of utrAP24-Gluc plants against the obligate biotrophic oomycete *P*. *hyoscyami* f. sp. *tabacina*, we carried out field trials during the season with high prevalence of tobacco blue mold, as described in^49^. Remarkably, utrAP24-Gluc 2 and 6 transplastomic plants showed a high level of resistance to *P*. *hyoscyami* f. sp. *tabacina* when compared with utrGus and wild-type control plants (Fig. 7C). At 35 days post-planting, we could observe clear disease symptoms in the utrGus and wild-type control plants. Control plants died as a result of disease progression while utrAP24-Gluc 2 and 6 transplastomic lines remained healthy (Fig. 7C). As previously observed in our experiments, disease resistance was associated with very low accumulation of pathogen biomass. Real-time PCR confirmed that a slight increase in biomass occurred in the utrAP24-Gluc 2 and 6 plants in contrast to what occurs in utrGus and wild-type control plants (Fig. 7D).

Field trial assay results further suggest that transplastomic tobacco plants transformed at the plastid genome level to express AP24 and β-1,3-glucanase in the chloroplast are resistant against a broad spectrum of phylogenetically diverse filamentous pathogens.

## Discussion

Several strategies have been evaluated to obtain disease resistant crops through genetic engineering of the nuclear genome, including overexpression of PR proteins coding genes (for a review see^17,29^). Although constitutive expression of PR proteins has been considered a promising strategy, limitations were observed, including low expression levels and the appearance of lesion mimic phenotypes^32,33^, have made it non-viable from a breeding perspective. In order to evaluate if tobacco PR proteins AP24 and β-1,3-glucanase can provide protection against filamentous pathogens without any associated undesired phenotypes when expressed from the plastid genome, we took advantage of plastid genome transformation of tobacco plants. Tobacco is the most important non-food crop worldwide. Its production is limited by filamentous pathogens (*Peronospora hyoscyami* f.sp *tabacina*, *Phytophthora nicotianae* and *R*. *solani*), and pests (especially *Heliothis virescens* and *M*. *persicae*)^50^. In addition, tobacco is a model species for studies in *Solanaceae* family, which includes members that are crucial as food sources, including potato, tomato and pepper. We could obtain three independent and homoplasmic transplastomic tobacco lines transformed at the plastid genome level with the *N*. *tabacum* AP24 and β-1,3-glucanase coding sequences. One of these lines, utrAP24-Gluc 7, showed unexpected profiles in transgene-specific PCR, Southern and northern blot assays (Figs 2 and 3), suggesting rearrangements in the plastid genome. For this reason, this line was not included in the infection assays. Tobacco transplastomic lines utrAP24-Gluc 2 and 6 showed expression of both transgenes AP24 and β-1,3-glucanase, and presented a phenotype indistinguishable from control plants (wild-type and transplastomic tobacco line utrGus). When these lines were challenged with the necrotrophic fungal pathogen *R*. *solani* under greenhouse conditions, they exhibited an outstanding resistant phenotype compared to control plants (Fig. 6). The same transplastomic tobacco lines AP24-Gluc 2 and 6 were evaluated to assess their performance under field conditions for infection with the oomycetes *P*. *hyoscyami* f.sp *tabacina* (biotroph) and *P*. *nicotianae* (hemibiotroph) (Fig. 7). Remarkably, utrAP24-Gluc plants showed a resistant phenotype against these two oomycete pathogens under high inoculums pressure as evidenced by a high disease incidence in the control plants. According to previous publications, AP24 (PR-5) and β-1,3-glucanase (PR-2), are implicated in defense responses against a diverse group of pathogens, including fungal and oomycete pathogens with different lifestyles^10,17^. For instance, Arabidopsis *cep* mutant with constitutive expression of PR-1, PR-2 and PR-5 showed enhanced resistance to *Peronospora parasitica*^51^. Transgenic plants transformed at the nuclear genome level to overexpress osmotin from different sources conferred variable degrees of protection against filamentous pathogens including *R*. *solani*^52,53^, *Botrytis cinerea*, *Oidium lycopersicum Leveillula taurica* and *Phytophthora infestans*^52,54^, *Sclerotinea sclerotiorum*^55^, *Fusarium pallidoroseum* and *Colletotrichum sp*.^56^. Protection against different filamentous pathogens in transgenic plants due to basic β-1,3-glucanase overexpression, has been observed for *R*. *solani*^57^, *Phytophthora infestans*^58^, *Fusarium sp*.^59^, *Cercospora arachidicola* and *Aspergillus flavus*^60^. A strategy based on the combination of multigene tolerance has been shown to improve the degree of resistance, as well as its durability. In some cases, co-expression of two or more PR proteins was able to provide an increased protection against filamentous pathogens in transgenic plants^24,61^. As observed from the literature, over-expressing osmotin or a β-1,3-glucanase has been proven to protect plants effectively against *R*. *solani*, but as far as we know, there is no report of their efficacy to control *P*. *hyoscyami* f.sp. *tabacina* or *P*. *nicotianae* in transgenic plants overexpressing these particular PR proteins.

The expected subcellular localization for endogenous β-1,3-glucanase and AP24 PR proteins are the vacuole and/or the apoplast^10,16,25^. Despite these proteins have been discovered more than 30 years ago, the molecular mechanism responsible for their antifungal activity is not well understood. In the case of β-1,3-glucanases, it has been proposed they exert their role in defense by two ways: they could act directly disrupting pathogen membrane/cell wall integrity^62^ or indirectly by releasing elicitors (like β-glucan) that in turn can trigger immunity in plants to activate plant defense responses^29,63,64^. For osmotin, different modes of action has been suggested^65^: disrupting fungal plasma membrane, interfering with signal transduction pathways in target pathogen cells, and releasing DAMPs in a similar way as β-1,3-glucanases. It has also been shown that osmotin triggers accumulation of the osmolyte Proline^65^, which in turn has been related to ROS balance and defense responses^66^. The different mechanisms mentioned above could explain the resistant phenotype observed for transplastomic utrAP24-Gluc plants in this work for *R*. *solani* (necrotroph) and *P*. *nicotianae* (hemibiotroph), since both PR proteins could potentially reach their targets once the plant cell integrity is compromised due to cell death and the plastid content is released. It has also been described that filamentous pathogens can secrete inhibitors affecting PR protein function during plant-pathogen interaction. For instance, *Phytophthora sojae* secretes a glucanase inhibitor proteins that can inhibit a soybean β-1,3-glucanase belonging to the PR-2 class^67,68^. Taking these observations into account, we cannot rule out that expression of PR-coding transgenes from the plastid genome confers resistance by allowing the host to store a pool of PR proteins that cannot be targeted by pathogen inhibitor molecules at specific time points during infection.

Of particular interest is the observation that expression of PR coding transgenes from the chloroplast genome can confer resistance to an obligate biotrophic pathogen, like *P*. *hyoscyami* f.sp *tabacina*. Although further experiments are required to determine the underlying mechanism responsible for this resistant phenotype, based on current knowledge we can propose some hypothetical ways for plastid material to get into contact with a biotrophic/hemibiotrophic pathogen. In the case of biotrophic and hemibiotrophic pathogens, cell integrity is maintained and very specialized structures called haustoria are formed at the interface of the interaction to allow pathogen nutrition^69^. It has been observed that during biotic stress, emergence of stromules (narrow tubular structures with stromal content) from plastids is enhanced^70,71^. It has been suggested that stromules could put in proximity plastid content with haustoria^72^, and might facilitate transmission of proteins and ROS from the main plastid body to other intracellular locations^70^. Moreover, several types of vesicles containing plastid material can arise from stromules and/or the main plastid body under physiological conditions^70,73^. Additionally, recent studies highlight the role of unconventional protein secretion (UPS) pathways in plant defense mechanisms (for a review see^74^). Preliminary results indicate that certain vesicles containing plastid material can localize around haustoria, where they might fuse to the plasma membrane and release their content into the extrahaustorial matrix (Tolga Bozkurt, personal communication). Further research is required to understand the molecular basis for the resistant phenotype observed in this work.

In this paper we have shown that transformation of the plastid genome with PR protein-coding genes confers a strong resistance phenotype against fungi and oomycete in transgenic plants both in greenhouse infection assays and under field inoculum pressure, without any noticeable effect on the phenotype. The fungi and oomycetes evaluated in this work include some of the most relevant plant pathogens responsible for enormous economic losses on crop species. Our experiments show that co-expression of AP24 and β-1,3-glucanase PR proteins by way of plastid genome transformation is a viable strategy to deploy disease resistance in crops.

**Table 1:** Primers used in this study.

Primer name	sequences (5′ to 3′)	Primer use
AP24-F	CTCATATGGCCACTATCGAGGTCC	Vector construction
AP24-R	GGTCTAGATTAACCATTAGGAC	
Gluc-F	GGATCACCCAAAGTTGATATTATATTTGG	Vector construction
Gluc-R	GCTCAATCGATAGGTGTTTGC	
T7UTR-F	GGAGACCACAACGGTTTCC	Vector construction
T7UTR-R	GTTATCGATTGCATATGTATATCTCC	
Fw1	GTATCTGGGGAATAAGCATCGG	PCR analysis
Rv1	CGATGACGCCAACTACCTCTG	
Fw2	CATACTTGAAGCTAGACAGG	PCR analysis
Rv2	CTCTACCACTGAGCTAATAGCC	
qPCR1-F	CTTCTCACCGAGGCTCCACT	qPCR
qPCR1-R	TGGAACCGTATGCGTCACTC	
qPCR2-F	TTCACATCCAGGGTGGTCAG	qPCR
qPCR2-R	CACGGACCGAGTCCATTGTA	
qPCR3-F	GCTGCTGGCATCTTTTTGCT	qPCR
qPCR3-R	TTCATCGATGTGCGAGCCTA	
qPCR4-F	CACGGACCAAGGAGTCTGACAT	qPCR
qPCR4-R	TCCCACCAATCAGCTTCCTTAC	

## Methods

### Transformation vectors

utrAP24-Gluc construct was designed to express mature AP24 (UniProtKB - P14170) and β-1,3-glucanase (UniProtKB - P15797) proteins through plastid genome transformation in tobacco, as previously described^47^. Briefly, AP24 and β-1,3-glucanase sequence were amplified by PCR with Pfu DNA Polymerase (Invitrogen, Carlsbad, CA, USA). As templates we use DNA from plasmids pHAP12 and pHGL21, respectively (kindly given by Dr. Lazaro Hernandez, CIGB Havana, Cuba). Oligonucleotides to amplify AP24 and β-1,3-glucanase coding sequences were designed to include NdeI and XbaI restriction sites for further cloning steps: AP24-F and AP24-R, and Gluc-F and Gluc-R (Table 1) respectively. Both AP24 and β-1,3-glucanase sequences were amplified without the amino-terminal signal peptide and the carboxyl-terminal peptide that are cleaved off during post-translational processing and during transport to the plant vacuole. The 5′ untranslated region from T7 phage gene 10 was amplified using primers T7UTR-F and T7UTR-R (Table 1). Amplification products were cloned into pZeRO-2 (Invitrogen). Gel purified AP24 NdeI/XbaI fragment was sub-cloned into the chloroplast transformation vector pBSW-utrGus, previously digested with NdeI/XbaI to replace *uidA* sequence^47^. pZeRO2-T7UTR was digested with SpeI and XhoI and cloned into pCR1.2 generated pCR1.2-T7UTR. Glucanase-coding sequence was obtained using restriction enzymes BamHI and ClaI, gel purified and cloned into pCR1.2-T7UTR, previously digested with ClaI and BglII. T7UTR/Glucanase was then released by enzymatic digestion with SpeI, gel purified and cloned into the chloroplast transformation vector pBSW-utrAP24, which was previously digested with XbaI. The final vector was designated utrAP24-Gluc (Fig. 1A), and the identity of the assembled elements was confirmed by sequencing.

### Tobacco plastid transformation

Plastid transformation was carried out as previously described^36,47,75^ using the PDS 1000/He biolistic particle delivery system (Bio-Rad Laboratories, Hercules, CA, USA). Briefly, fully expanded leaves of *in vitro* cultured *N*. *tabacum* L. cv. Petit Havana plants were bombarded with 50 mg of 0.6-µm gold particles (Bio-Rad) coated with 10 µg of plasmid DNA using 1,100 psi rupture discs (Bio-Rad). Spectinomycin-resistant lines were selected on RMOP regeneration medium^75^ containing 500 mg L^-1^ spectinomycin di-hydrochloride. Three independent transplastomic lines were selected and subjected to subsequent regeneration rounds on selective medium to obtain homoplasmic lines. Plants were acclimated to greenhouse conditions under the following parameters: natural light was supplemented 16 h per day by sodium lamps; the temperature was set at 26 °C during day and 19 °C in the night.

### PCR analysis to confirm transplastomic nature and construct integrity

Integration of transgenes in the plastid genome was confirmed by PCR amplification using as template total leaf DNA obtained from regenerated shoots and wild-type plants, as previously described^47,76^. To analyze the transplastomic nature of regenerated shoots we used primers Fw1 and Rv1 (Table 1, Fig. 1); a 1450 bp fragment is only amplified when transgenes are integrated in the plastid genome at the desired region (Fig. 1). Another PCR reaction was performed in order to evaluate the construct integrity, using primers Fw2 and Rv2, which anneal within the integrated cassette (Table 1). In this case, a 2440 bp fragment was only amplified from transplastomic plants with intact transgenes. PCR reactions were conducted as previously described^47,76^, with a differential annealing temperature of 50 °C for Fw2 + Rv2 PCR reaction.

### Southern blot analysis

Southern blot was performed as previously described^47^. Briefly, 3 µg of total leaf DNA digested with NcoI (New England Biolabs, Beverly, MA, USA) were electrophoresed in 0.8% agarose gel, and blotted onto HybondN+ Nylon membrane (Amersham Biosciences, Uppsala, Sweden). The membrane was hybridized with a ^32^P-labeled trnI/A DNA probe, generated by random priming with a Prime-a-Gene kit (Promega, Madison, WI, USA). Pre-hybridization and hybridization were carried out following published protocols^47,77^. The blot was analyzed after exposure to a storage phosphor screen, using a Storm 840 PhosphorImager system (Amersham Biosciences).

### Northern blot analysis

Total leaf RNA extracted using TRiZOL Reagent (Invitrogen) was analyzed by northern blot as previously described^36,47^. Briefly, 5 µg of denatured RNA prepared in formaldehyde containing buffer were separated by electrophoresis in a 1.5% agarose/formaldehyde gel. After blotting onto HybondN+ Nylon membranes (Amersham Biosciences), RNAs of interest were identified using ^32^P-labeled DNA probes (AP24 or glucanase) synthetized by random priming with a Prime-a-Gene kit (Promega). Pre-hybridization, hybridization and washing were performed as described in^47^.

### Protein extraction and analysis

Total protein extracts were obtained by processing 25 mg of leaf tissue in 125 μL of Laemmli^78^ sample buffer. For SDS-PAGE, 20 μL of each extract were electrophoresed in 15% gels and transferred onto nitrocellulose membrane for later antibody detection. β-1,3-glucanase protein was detected using a PR-2 specific rabbit polyclonal antibody (Agrisera, Vännäs, Sweden) as previously described^79^. AP24 protein was detected using a specific rabbit antiserum for AP24 (kindly provided by Dr. Lázaro Hernandez from CIGB, Havana, Cuba), as previously described^80^. In both cases, the secondary antibody used was a 1:3000 dilution of a goat anti-rabbit antibody conjugated to calf intestinal alkaline phosphatase (Cell Signaling Technology, Danvers, MA, USA). To detect phosphatase activity, 5-bromo-4 chloro-3 indolyl phosphate and nitroblue tetrazolium (Sigma, St. Louis, MO, USA) were used as substrates.

### Inoculation with *Rhizoctonia solani*

Resistance to *R*. *solani* was evaluated as previously described^49^. An aggressive isolate of *R*. *solani* (anastomosis group n. 3) was used for inoculations. Agar-mycelium plugs from 5 days cultures grown on potato dextrose agar (22–25 °C) were used to colonize rice grains for approximately 2 weeks at room temperature. Two-week-old tobacco seedlings (90 seedlings for each transplastomic line or wild-type plants) were inoculated with approximately six *R*. *solani* colonized rice grains placed onto the soil surface^81^. At 3 weeks post-inoculation (wpi), disease symptoms were evaluated visually and pathogen growth on inoculated plants was measured by quantitative real-time reverse transcription PCR^49^ using primers qPCR1-F and qPCR1-R to amplify *R*. *solani* beta actin sequence (Table 1). Amplification products were sequenced to confirm their identity (Accession: KC285905.1). Disease incidence (percentage of plants exhibiting symptoms) was evaluated after 3 wpi^49^. Data was analysed as described in^82^. To improve homogeneity of variance, all percent incidence data were subjected to an arcsine transformation before statistical analysis; data was then analyzed by one-way analysis of variance using GraphPad Prism Software Inc. (La Jolla, CA, USA). Significant difference among mean values was determined by Tukey’s Multiple Comparison Test at P < 0.0001.

### Resistance to *Phytophthora nicotianae*

Resistance to *P*. *nicotianae* was evaluated as previously described^49^. To determine the performance of tobacco plants under field conditions, trials were conducted in experimental fields with a high incidence of *P*. *nicotianae* located at the Center of Biotechnology and Genetic Engineering, Havana, Cuba. Transplastomic lines and control plants were evaluated by planting ninety two-week-old plants of each line following a random design. At ten days post-planting, development of stem lesions was visually evaluated using the linear scale of 1–10 (being 1 no disease and 10 plant death) as previously described^83^. At the same time point, growth of *P*. *nicotianae* on tobacco plants was estimated by quantitative real-time PCR using the primers qPCR2-F and qPCR2-R (Table 1). Amplification products were sequenced to confirm their identity (*P*. *nicotianae* sequence, Accession: DQ059571.1). Data was analyzed by one-way analysis of variance using GraphPad Prism Software Inc. (La Jolla, CA, USA)^82^. Significant difference among mean values was determined by Tukey’s Multiple Comparison Test at P < 0.0001.

### Resistance to *Peronospora hyoscyami* f. sp. *tabacina*

Resistance to *P*. *hyoscyami* f. sp. *tabacina* was evaluated as previously described^49^. To determine the performance of tobacco plants under field conditions, trials were conducted in experimental fields located at the Center of Biotechnology and Genetic Engineering, Havana, Cuba, where *P*. *hyoscyami* f. sp. *tabacina* is a significant problem for tobacco production each year. During cold and wet season, transplastomic lines and control plants were evaluated by planting ninety 8-week-old plants of each line in the field following a random design; five replicates were made. At 35 days post-planting, plants were visually evaluated to determine the percentage of healthy plants (plants showing up to three blue molds spots per leaf)^49^. Additionally, growth of *P*. *hyoscyami* f. sp. *tabacina* was analysed by quantitative real-time PCR^49^, using primers qPCR3-F and qPCR3-R to amplify the constitutively expressed 18S ribosomal RNA gene (Table 1). PCR products were sequenced to confirm their identity (*P*. *hyoscyami* f. sp. *tabacina*, Accession: DQ067899.1). Data was analysed as described in^82^. To improve homogeneity of variance, all percent data were subjected to an arcsine transformation before statistical analysis; data was then analysed by one-way analysis of variance using GraphPad Prism Software Inc. (La Jolla, CA, USA). Significant difference among mean values was determined by Tukey’s Multiple Comparison Test at P < 0.0001.

### RNA extraction and quantification of fungal and oomycete biomass

Plant samples (500 mg of tissue pooled from leaves, stems and roots) collected from 10 plants for each control and transplastomic line were immediately placed in liquid nitrogen and processed for RNA extraction and cDNA synthesis as previously described^84^. For real time PCR, the QuantiTect SYBR Green PCR Kit (Qiagen, Maryland, USA) was used for relative quantification and reactions were performed in a Rotor - Gene 3000 PCR machine (Corbett, Australia) following reported reaction setup^84^. For fungal and oomycete biomass determination primer sequences (Table 1) were designed based on Primer 3 online software (http://bioinfo.ut.ee/primer3/). Each cDNA sample was evaluated by real time PCR in triplicates. The extent of colonization was determined as described in^49^, by the relative quantification of transcript levels of the constitutively expressed pathogen genes to the constitutively expressed tobacco 26S rRNA gene (primers qPCR4-F and qPCR4-R, Table 1). Bars indicate mean values and standard errors of three samples taken from ten plants for each point analyzed. The relative levels of genes were expressed as ‘Mean Normalized Expression’ using Q-Gene software (http://www.gene-quantification.de/qgene.zip)^85^. Data was analysed as previously described^83^, by two-way ANOVA analysis of variance using GraphPad Prism Software Inc. (La Jolla, CA, USA). Significant difference among mean values was determined by Bonferroni multiple comparisons Test at P < 0.0001.

## Supplementary information

Supplementary Information

## Data Availability

All data generated or analysed during this study are included in this published article.
