# Author Correction: Expression of pathogenesis-related proteins in transplastomic tobacco plants confers resistance to filamentous pathogens under field trials

**DOI:** 10.1038/s41598-021-95177-2

**Published:** 2021-08-05

**Authors:** Noelia Ayelen Boccardo, María Eugenia Segretin, Ingrid Hernandez, Federico Gabriel Mirkin, Osmani Chacón, Yunior Lopez, Orlando Borrás-Hidalgo, Fernando Félix Bravo-Almonacid

**Affiliations:** 1 Laboratorio de Biotecnología Vegetal, Instituto de Investigaciones en Ingeniería Genética y Biología Molecular “Dr. Héctor N. Torres” (INGEBI-CONICET), (C1428ADN) Ciudad Autónoma de Buenos Aires, Argentina; 2 Departamento de Fisiología, Biología Molecular y Celular, Facultad de Ciencias Exactas y Naturales, Universidad de Buenos Aires, (C1428EGA) Ciudad Autónoma de Buenos Aires, Argentina; 3 Centro de Ingeniería Genética y Biotecnología (CIGB), (10600) La Habana, Cuba; 4 Shandong Provincial Key Laboratory of Microbial Engineering, School of Biotechnology, Qi Lu University of Technology, Jinan, (250353) P. R. China; 5 Departamento de Ciencia y Tecnología, Universidad Nacional de Quilmes, Bernal, (B1876BXD) Buenos Aires, Argentina

Correction to: *Scientific Reports* 10.1038/s41598-019-39568-6, published online 26 February 2019

In the original version of this Article, Fernando Félix Bravo-Almonacid was omitted as a corresponding author. Correspondence and request for materials should also be addressed to fbravo@dna.uba.ar.

This error has now been corrected in the HTML and PDF versions of the Article.

