# Supplementary material for: Expression of pathogenesis-related proteins in transplastomic tobacco plants confers resistance to filamentous pathogens under field trials
Source: Sci Rep. 2019 Feb 26;9:2791. doi: 10.1038/s41598-019-39568-6 (PMC6391382; doi:10.1038/s41598-019-39568-6)
Supplement: Supplementary file 1 — Supplementary Information [file 41598_2019_39568_MOESM1_ESM.pdf]

Expression of pathogenesis-related proteins in transplastomic tobacco plants confers resistance to filamentous pathogens under field trials

Noelia Ayelen Boccardo1#, María Eugenia Segretin1,2*#, Ingrid Hernandez3, Federico Gabriel Mirkin1, Osmani Chacón3, Yunior Lopez3, Orlando Borrás-Hidalgo3,4 and Fernando Félix Bravo-Almonacid1,5

[1]Laboratorio de Biotecnología Vegetal, Instituto de Investigaciones en Ingeniería Genética y Biología Molecular (INGEBI-CONICET), Ciudad Autónoma de Buenos Aires (C1428ADN), Argentina

[2]Departamento de Fisiología, Biología Molecular y Celular, Facultad de Ciencias Exactas y Naturales, Universidad de Buenos Aires, Ciudad Autónoma de Buenos Aires (C1428EGA), Argentina

[3]Centro de Ingeniería Genética y Biotecnología (CIGB), La Habana (10600), Cuba

[4]Shandong Provincial Key Laboratory of Microbial Engineering, School of Biotechnology, Qi Lu University of Technology, Jinan (250353), P.R. China

[5]Departamento de Ciencia y Tecnología, Universidad Nacional de Quilmes, Bernal, Buenos Aires (B1876BXD), Argentina

[#] Noelia Ayelen Boccardo and María Eugenia Segretin contributed equally to this work.

* Corresponding author: correspondence and requests for materials should be addressed to María Eugenia Segretin (email: segretin@dna.uba.ar)

Supplementary information

Figure S1

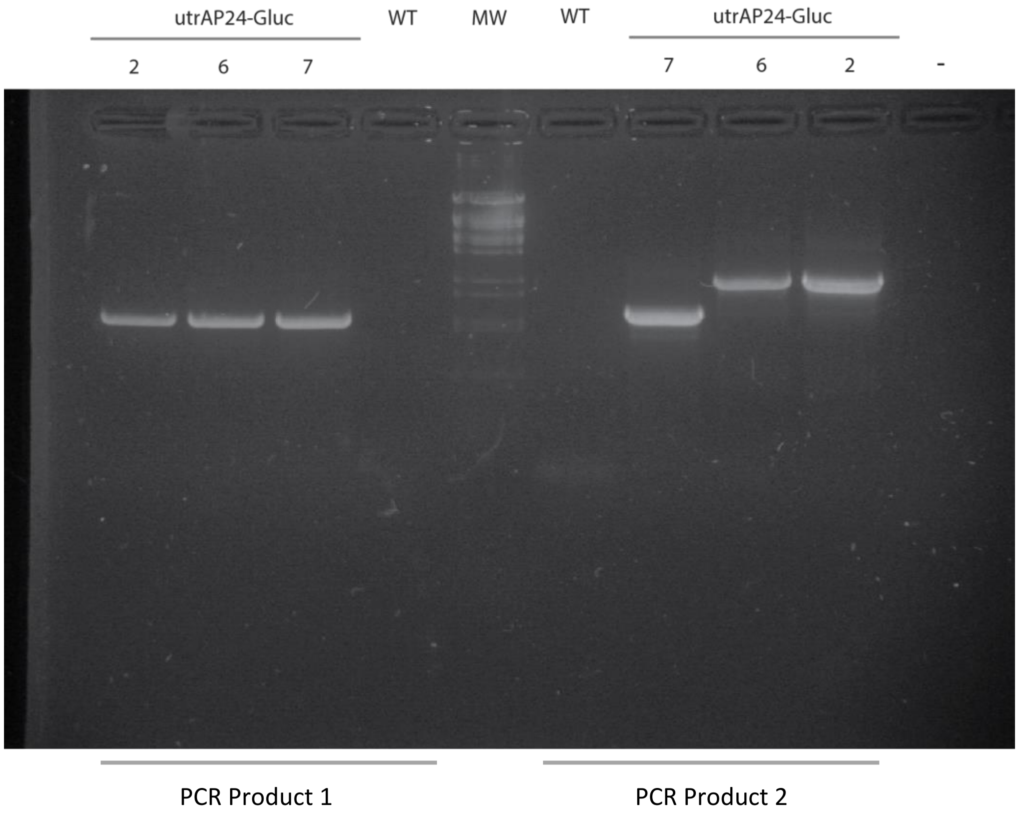

Supporting information for Figure 2: "Analysis of transgene integration and homoplasmic state". PCR analysis to confirm integration of the recombinant vector into the wild-type plastome, using Fw1 and Rv1 (PCR product 1) and Fw2 and Rv2 primers (PCR product 2). WT: wild-type tobacco plant. MW: Lambda BstEII marker. This image corresponds to the full size image from where Figure 2A was prepared.

Figure S2

A

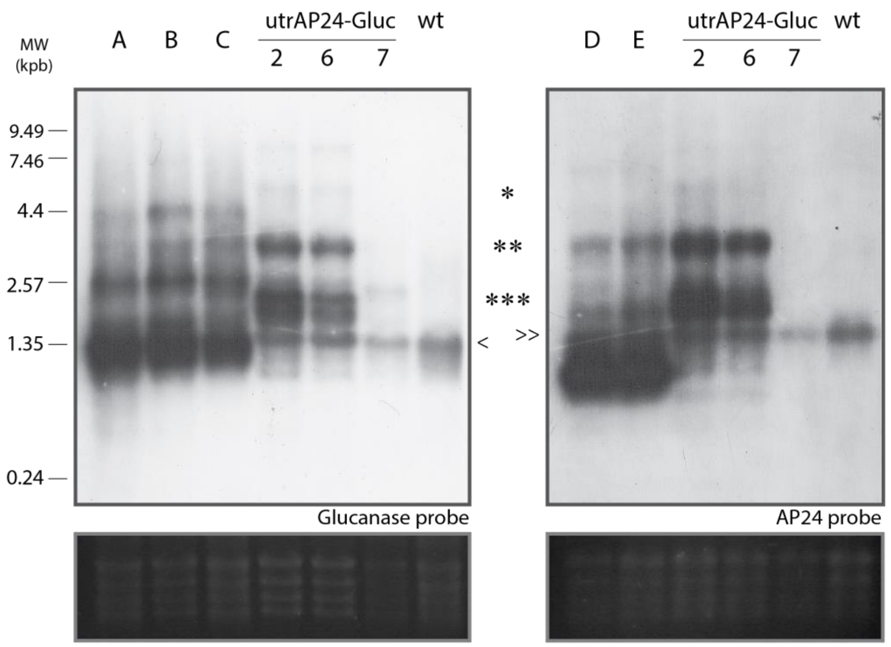

B

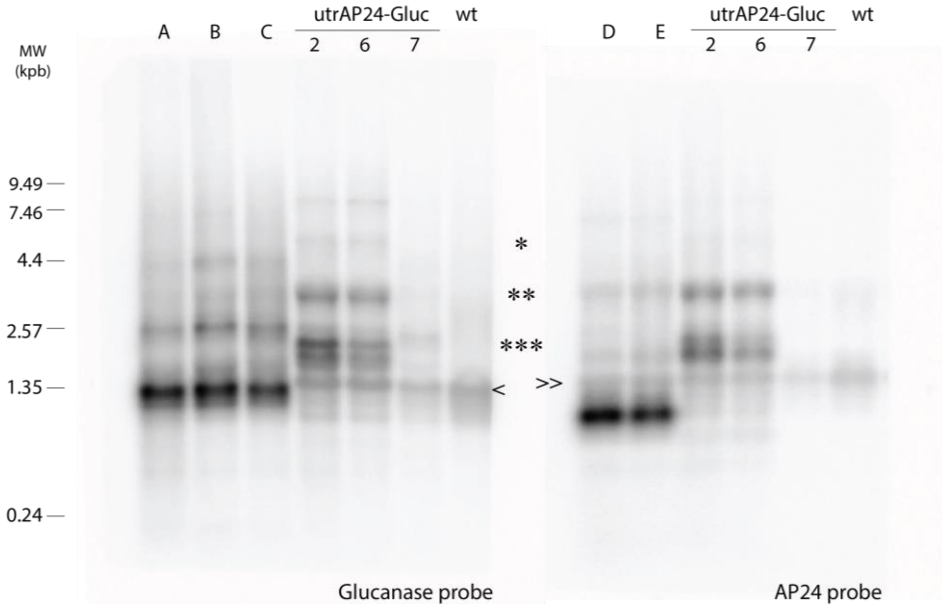

Supporting information for Figure 3: "Characterization of transcripts containing the glucanase and AP24 sequence of transplastomic plants". (A) Northern blot using glucanase (left panel) and AP24 (right panel) probes showing transcript generation. These images correspond to the scanned autoradiographs of full size blots for each probe, using autoradiography films (GE Healthcare Amersham). Samples A, B, C, D and E correspond to transgenic plants not related to this work. Transcripts observed for utrAP24-Gluc plants: *, policistronic; **, tricistronic; ***, dicistronic; and endogenous glucanase and AP24 (< and >>, respectively). Below: Total rRNA as observed under UV light was included as loading control. **(B)** Images correspond to the same full size blots as in (A) after a reduced exposure using a BAS Storage Phosphor Screen (Fujifilm).
